# Psychogenic Equinovarus Caused by Dislocation of the Chopart Joint Complex

**DOI:** 10.1155/2018/2736917

**Published:** 2018-05-03

**Authors:** Ichiro Tonogai, Koichi Sairyo

**Affiliations:** Department of Orthopedics, Institute of Biomedical Science, Tokushima University Graduate School, 3-18-15 Kuramoto, Tokushima 770-8503, Japan

## Abstract

Patients with conversion disorder (CD) present with weakness or unexplained movement disorder that may evolve from inciting psychological events, but presentation with rigid deformity is rare. Only one case of CD presenting as foot deformity with atraumatic rigid psychogenic equinovarus has been reported previously. Here we describe a rare case of psychogenic equinovarus in a physically healthy 10-year-old boy. He had noticed left equinovarus deformity upon waking abruptly but had no history of preceding trauma and no relevant medical history. Computed tomography (CT) images revealed dislocation of the left Chopart joint complex, but clinical examination did not suggest an organic neurologic disorder. On further history taking, he reported that he was under psychological stress because of being required to play baseball against his will. When he was given permission to withdraw from this stressful situation, the equinovarus improved without the need for surgical invention. This report highlights the importance of early and accurate diagnosis of psychogenic equinovarus, so that unnecessary surgery can be avoided. This is the first report of psychogenic equinovarus caused by dislocation of the Chopart joint complex that was confirmed with CT.

## 1. Introduction

Conversion disorder (CD) is essentially a psychogenic condition with symptoms that cannot be traced to an identifiable organic disorder and are incompatible with known anatomy and physiology [1]. Symptoms of CD are not generally feigned but are an involuntary manifestation of psychological conflict [2]. The prevalence of CD in children has been estimated at 2–4 per 1,00,000 [3] and in one series it was reported that 22% of children underwent unnecessary surgery before an accurate diagnosis of CD was made [4].

Rigidity and contractures are rare in CD [5, 6] and reports of deformities have been limited to the upper extremity [7]. There has been a previous report of CD manifesting as foot deformity with rigid psychogenic equinovarus in the absence of trauma [8]. However, there has never been a report of psychogenic equinovarus caused by dislocation of the Chopart joint complex (the articulation between the talonavicular and calcaneocuboid joints) confirmed on computed tomography (CT) images.

In this report, we describe a rare case of a physically healthy 10-year-old boy who presented with rigid equinovarus caused by dislocation of the Chopart joint complex in association with psychological stress that was diagnosed early and improved spontaneously without surgical invention.

## 2. Case Report

A physically healthy 10-year-old boy, who weighed 32 kg (just below 50 percentile) at a height of 1.34 m (just above 50 percentile), noticed sudden atraumatic left foot deformity on waking up in the morning. There was no relevant past medical history. A visit to a local doctor was arranged promptly, and he was referred to our department for further examination and treatment within 2 days.

**Figure 1:**
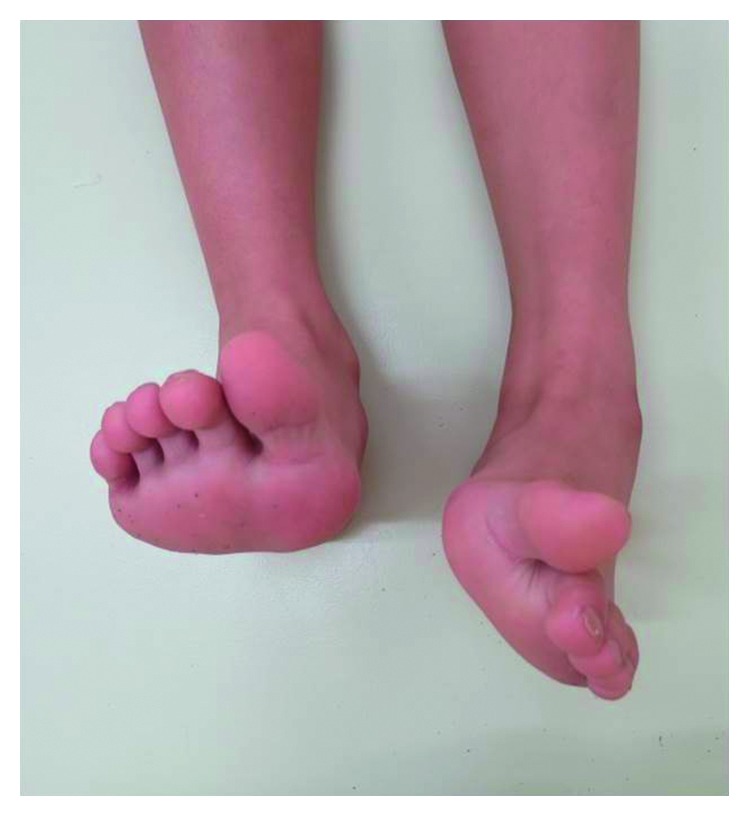
A photograph showing uncorrectable equinovarus deformity of the left foot on nonweight-bearing frontal view.

**Figure 2:**
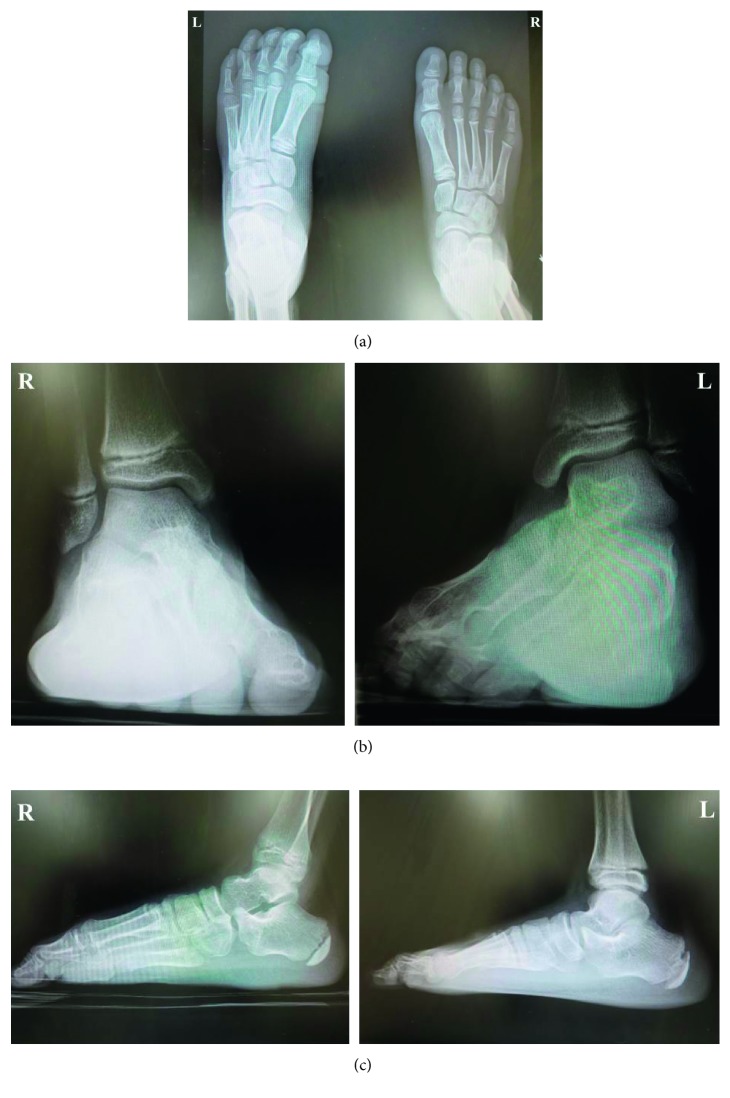
Plain weight-bearing radiographs reveal inversion of the left forefoot on (a) dorsoplantar view and (b) anteroposterior view. (c) Lateral view showing incongruity of the Chopart joint complex.

**Figure 3:**
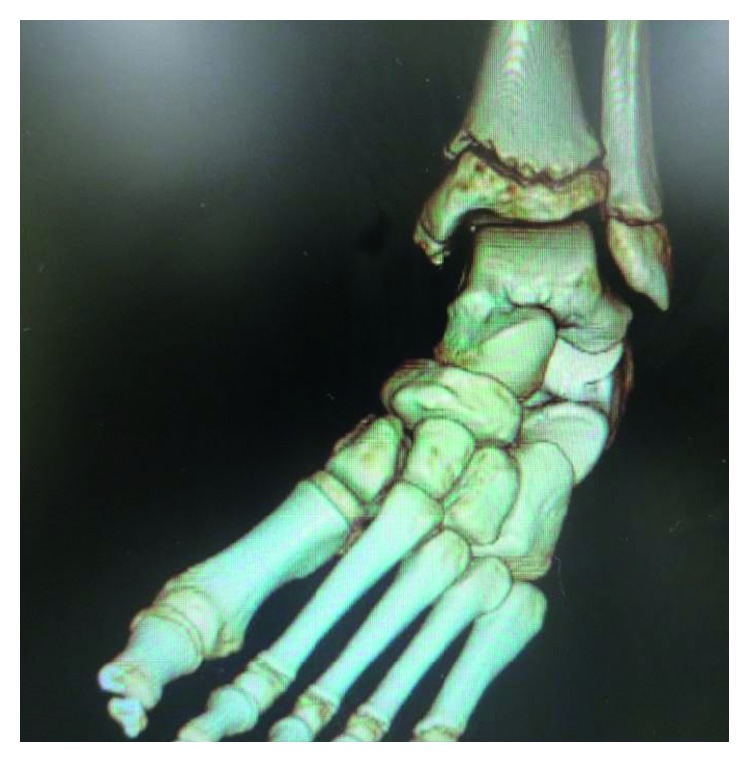
Dislocation of the left Chopart joint complex can be seen on the frontal computed tomography image.

On physical examination, there was slight tenderness and swelling over the dorsal aspect of the left midfoot. The left forefoot was rigid and held in an equinovarus position (Figure 1). Almost all of the load was transmitted through the lateral border of the left foot. Muscle strength was intact in the left toe extensors and flexors, and there was no sensory disturbance over his left foot. Abnormal body movements, such as tremor, clonus, tics, or dystonia, were not detected. Plain radiographs showed inversion of the left forefoot (Figures 2(a) and 2(b)), suggesting incongruity of the Chopart joint complex (Figure 2(c)), which was confirmed by CT (Figure 3). He was then referred to the Department of Clinical Neuroscience in our hospital. Blood laboratory results were within normal limits, and magnetic resonance images of the brain revealed no organic abnormality. There was no evidence of neurological disorder on clinical examination.

Detailed history taking revealed that the boy had been under psychological stress. He had been playing baseball but was doing so reluctantly to please his mother. He wanted to tell his mother and the baseball team coach of his wish to quit baseball but felt unable to do so. Furthermore, his parents were divorced, and his father had remarried and started a second family. Considering all the events in his social history, we concluded his foot deformity was psychogenic equinovarus accompanied by CD. Therefore, we opted for conservative treatment rather than surgical reduction under general anesthesia.

When the boy woke up on the morning after visiting our hospital, the left rigid equinovarus deformity had disappeared. However, it returned 2 days later, and then resolved completely after another 2 days. Subsequently, the clinical course was uneventful, with no return of the fixed deformity after he was allowed to quit playing baseball. He continues to have no pain or discomfort in his left foot and has no limitations on physical activity.

## 3. Discussion

CD is much more likely to involve paralysis or weakness than rigidity or contracture [5, 6]. There has been only one report of atraumatic rigid psychogenic equinovarus accompanied by CD to date [8]. To our knowledge, the present report is the first of rigid psychogenic equinovarus confirmed on CT images that was caused by dislocation of the Chopart joint complex in a patient in whom physical symptoms were associated with psychological stressors and could not be explained by organic pathology. Our case suggests that psychogenic equinovarus can occur at the level of the Chopart joint complex.

Women are 2–10 times more likely to develop CD than men [9], and the dominant extremities are more likely to be involved than the nondominant extremities [10, 11]. However, our patient was male and his equinovarus deformity occurred on the nondominant side.

Symptoms of CD are usually self-limiting and do not lead to any lasting disability, although patients with psychogenic tremors or seizures, concomitant medical illness, or pending litigation issues generally have a less favorable prognosis [12–14]. In our patient, where onset was sudden (overnight), symptoms were of short duration, he was in previous good health, and had no coexisting organic psychopathology but had an identifiable stressor, the clinical outcome was good.

In one series, 22% of children were reported to have undergone surgery needlessly before their symptoms were accurately diagnosed as being related to CD [4]. Although accurate diagnosis is challenging in such patients, careful history taking and physical examination can often unmask the presence of psychological conflict and avoid unnecessary surgery. In the event of recalcitrant symptoms, a combination of aggressive physical therapy and psychotherapy might be necessary [15–17].

As for diagnosis of psychogenic equinevarus, although neurologic examinations including electromyography are usually within normal limits [1, 12, 16], they may be useful to rule out other known diseases. However, having enough knowledge of neurophysiology and neuroanatomy may be the most important to make a diagnosis of psychogenic equinevarus, when symptoms and sings are inconsistent with known disease processes and cannot be explained by any known anatomic neurological lesion. It may be also important to recognize that psychogenic equinovarus is a conversion reaction which is an unconscious process that should not be confused with malingering although it may reflect an attempt to avoid external stresses such as psychological conflict.

A limitation of this case report is its short follow-up duration. Careful longer-term follow-up of other cases would be necessary in the future. Another limitation is the name of CD in *Diagnostic and Statistical Manual of Mental Disorders*, Fourth Edition (DSM-4). Although that name refers to the hypothesis based on psychoanalytic etiology and has not been accepted by either nonpsychiatrists or patients [18, 19]. Therefore, as Stone et al. and Cosci et al. suggested, we might rename CD “functional neurological symptom disorder” according to DSM, Fifth Edition (DSM-5) [19, 20].

In conclusion, we encountered a rare case of rigid psychogenic equinovarus in a physically healthy 10-year-old boy. The patient interview, including a psychiatric consultant, revealed underlying psychological stress and led to an early and accurate diagnosis. Therefore, this deformity was successfully managed conservatively without surgical invention. While not overlooking the possibility of coexistent organic disease, we should keep in mind psychological conflict as a potential cause of conversion reactions.
